# Serum and Serum Exosomal CircRNAs hsa_circ_0001492, hsa_circ_0001439, and hsa_circ_0000896 as Diagnostic Biomarkers for Lung Adenocarcinoma

**DOI:** 10.3389/fonc.2022.912246

**Published:** 2022-06-07

**Authors:** Yanli Kang, Jianbin You, Yuhan Gan, Qianshun Chen, Chen Huang, Falin Chen, Xunyu Xu, Liangyuan Chen

**Affiliations:** ^1^ Department of Clinical Laboratory, Fujian Provincial hospital, Shengli Clinical Medical College of Fujian Medical University, Fuzhou, China; ^2^ Department of Thoracic Surgery, Fujian Provincial hospital, Shengli Clinical Medical College of Fujian Medical University, Fuzhou, China

**Keywords:** exosome, lung adenocarcinoma, biomarkers, circRNA, serum

## Abstract

**Background:**

Circular RNAs (circRNAs) play an important role in tumorigenesis and several circulating circRNA signatures are closely associated with tumor diagnosis. However, the expression and clinical significance of the two forms of circulating circRNAs, serum and serum exosomal, in patients with lung adenocarcinoma (LUAD), have not been characterized.

**Methods:**

Three differentially expressed exosomal circRNAs, hsa_circ_0001492, hsa_circ_0001439, and hsa_circ_0000896, were selected based on previous exosomal circRNA sequencing data analyses of LUAD patients. The expression of these circRNAs in serum and serum-derived exosomes of LUAD patients was assessed using quantitative real-time PCR (qRT-PCR), and correlations between circRNA expression and clinicopathological characteristics were analyzed. The reliability of serum and serum exosomal hsa_circ_0001492, hsa_circ_0001439, and hsa_circ_0000896 to diagnose LUAD was evaluated using receiver operating characteristic (ROC) analysis.

**Results:**

Expression of serum and serum exosomal hsa_circ_0001492, hsa_circ_0001439, and hsa_circ_0000896 were significantly higher in LUAD patients than in healthy donors, and significantly lower after surgery. These three serum exosomal circRNAs were also associated with a higher cancer stage. Exosomal hsa_circ_0001492 expression was positively correlated with carcinoembryonic antigen (CEA) and neuron-specific enolase (NSE) levels. An association between the expression of the three serum circRNAs and clinical characteristics was not observed. In addition, the three serum exosomal circRNAs had higher diagnostic sensitivity and specificity than the serum circRNAs, and the area under the curve (AUC) of all three serum exosomal circRNAs was >0.75. The combination of exosomal hsa_circ_0001492, hsa_circ_0001439, and hsa_circ_0000896 had better diagnostic sensitivity and specificity than that of a single marker, with an AUC value of 0.805.

**Conclusions:**

The serum and serum exosomal circRNAs, hsa_circ_0001492, hsa_circ_0001439, and hsa_circ_0000896, were upregulated in LUAD patients. Serum exosomal circRNAs may serve as more effective biomarkers than serum circRNAs for LUAD diagnosis and may further aid the detection of this disease.

## Introduction

Lung cancer is associated with high morbidity and mortality and continues to be a major public health problem worldwide (1). Lung adenocarcinoma (LUAD) is the most common histological type of lung cancer. While the 5-year survival rates of patients with lung cancer are still <15%, rates are closer to 50%–70% for non-small-cell lung cancer (NSCLC) patients who receive early-stage surgical resections (2). Lung cancer is usually diagnosed by X-ray, low-dose computerized tomography (CT) scan, and auxiliary tumor markers such as neuron-specific enolase (NSE) and carcinoembryonic antigen (CEA). X-ray and low-dose CT may result in false positives, however, increasing the risk of overdiagnosis (3). Unfortunately, because the early symptoms of lung cancer are subtle and easily overlooked, and there is a lack of specific diagnostic biomarkers, most patients are diagnosed in the advanced stages of the disease. Thus, there is an urgent need for the development of reliable diagnostic and therapeutic molecular biomarkers for lung cancers such as LUAD.

In recent years, circular RNAs (circRNAs) and exosomes have shown promise as biomarkers for various cancers. CircRNA, a large group of RNAs that form covalently closed continuous loops without protein-coding ability, was first discovered in an RNA virus in 1976 (4, 5). Particular attention has been focused on circRNAs because they are present and more stable in tissues, cells, and bodily fluids than linear RNA and are expressed in a tissue- and developmental stage-specific manner. CircRNAs can participate in physiological and pathological processes by regulating transcription, translation, and miRNA or protein sponges (6). CircRNAs play a vital role in tumor progression and show promise as cancer biomarkers. Hsa_circ_0004015, for example, regulates the proliferation, invasion, and TKI drug resistance of NSCLC through miR-1183/PDPK1 signaling while hsa_circ_001783 is a novel prognostic marker for breast cancer and regulates cancer proliferation and metastasis by sponging miR-200c-3p (7, 8).

Exosomes are extracellular vesicles that are 30-150 nm in diameter and contain bioactive molecules, such as nucleic acids (DNA, mRNA, microRNA, and other non-coding RNAs), proteins, and lipids (9). Studies indicate that tumor cells, especially those associated with ovarian, lung, colorectal, and breast cancer, secrete many more exosomes into the microenvironment than normal cells (10). The presence of circRNAs in tumor-related serum exosomes has received a great deal of attention in recent years. Since exosomes protect their cargoes from degradation, serum exosomal circRNAs, which have higher stability than serum circRNAs, can be powerful non-invasive diagnostic and prognostic tools for cancer (11, 12). Thus, it is worth exploring whether serum exosomal circRNAs may be more effective than serum circRNAs as biomarkers of early-stage cancers such as LUAD.

We have previously shown circRNA expression profiles in exosomes from early-stage lung adenocarcinoma and suggested that altered exosomal circRNA expression may aid the novel diagnosis and treatment of LUAD (13). Hsa_circ_0001439, hsa_circ_0001492, and hsa_circ_0000896 were shown to resist digestion by RNase R exonuclease and exist in a circular form with a back-splice junction. In this study, we further investigated the serum and serum exosomal expression of hsa_circ_0001439, hsa_circ_0001492, and hsa_circ_0000896 in LUAD and assessed their clinical value as biomarkers for the prognosis and diagnosis of this disease.

## Materials and Methods

### Clinical Patients

All LUAD patients were enrolled at the Fujian Provincial Hospital (Fuzhou, China). Patients were excluded from the study if they had diabetes mellitus, pregnancy, arteriosclerosis, severe liver and kidney disease, or another systemic illness, or had received chemotherapy, immunotherapy, or radiotherapy prior to blood collection. All serum samples were randomly separated to evaluate the expression of serum exosomal circRNAs (134 LUAD cases and 50 controls) and serum circRNAs (74 LUAD cases and 40 controls). Serum was collected from each patient before and on the 5th day after surgery. Clinicopathological data, including age, gender, tumor size, and TNM stage, were collected for all LUAD patients. This study was approved by the Ethics Committee of Fujian Provincial Hospital (K-2021-040-04), and all patients provided written informed consent.

### Serum Preparation

Peripheral blood (2–3 mL) collected from LUAD patients and healthy controls was stored at 4°C within 1 hour after collection. Samples were centrifuged at 2000 rpm for 10 min, transferred to RNase-free centrifuge tubes, and stored at -80°C for further analyses.

### Exosome Isolation

Enrichment and purification of plasma exosomes were conducted using the exoRNeasy Midi Kit (77144, Qiagen, Germany). Exosomes were isolated and purified from 1 mL serum according to the manufacturer’s instructions. In brief, 1mL serum was mixed with Buffer XBP and bound to an exoEasy membrane affinity spin column. The bound exosomes were washed with Buffer XWP and lysed with QIAzol. Detailed protocols are documented in www.qiagen.com/HB-1179. The pelleted exosomes were resuspended in 1x phosphate‐buffered saline (PBS), and their concentrations were estimated using the Bradford assay. All exosomes were stored at -80°C.

### Characterization and Determination of Exosomes

Western blotting, transmission electron microscopy (TEM), and nanoparticle tracking analysis (NTA) were used to observe the morphology and concentration of exosomes isolated from serum. A drop of this solution was added to a copper‐coated grid and incubated at room temperature for 5 min. Exosomes were negatively stained with 2% aqueous uranyl acetate for 2 min and washed three times in PBS. The exosomes were dried at room temperature and viewed by transmission electron microscopy (TEM) (HT-7700, Hitachi, Japan). Exosome pellets were diluted in PBS and the particle concentrations and distribution were analyzed using nanoparticle tracking analysis (NTA) (N30E, NanoFCM, China). Expression of the exosomal markers, CD9, CD81, and syntenin, were analyzed by Western blotting. Separate equivalent amounts of protein were separated using 10% sodium dodecyl sulfate polyacrylamide (SDS-PAGE) gel electrophoresis and the target proteins were transferred onto a polyvinylidene difluoride membrane. Rabbit polyclonal CD9 (Abcam, ab92726), CD81 (Abcam, ab109201), and syntenin antibody (1:1000 dilution; Abcam, ab19903) and secondary antibody (1:5000 dilution; Abcam) were used.

### Serum and Serum Exosomal RNA Extraction

Total RNA in 0.2 mL serum was extracted using TRIzol LS reagent (Invitrogen, Germany) according to the manufacturer’s instructions. Exosomal RNA was isolated from 1 mL serum using the exoRNeasy Serum Maxi Kit (Qiagen, Germany) according to the manufacturer’s instructions. The RNA concentration was determined using a NanoDrop spectrophotometer (NanoDrop™ One, Thermo Fisher Scientific, Waltham, MA, USA).

### CircRNA Detection by Quantitative Real-time Polymerase Chain Reaction

Equal amounts of RNA were reverse transcribed with random primers using the PrimeScript RT reagent Kit (TaKaRa, Dalian, China). Quantitative real-time PCR (qRT-PCR) of synthesized complementary DNA (cDNA) was performed with a LightCycler480 real-time quantitative PCR system (Roche, Shanghai, China) using TB Green^®^ Premix Ex Taq™ II (TaKaRa, Dalian, China) according to the manufacturer’s instructions. Each reaction was performed in triplicate and relative expressions were measured using the 2^−ΔΔCt^ or 2^−ΔCt^ method. Importantly, relative expressions in preoperative and postoperative LUAD patients were determined using the 2−^Δ^Ct method. Primers for circRNA were synthesized by SunYa Biotechnology (SunYa, Fuzhou, China). Details of the primer sequences used for qRT-PCR are shown in **Table 1**.

**Table 1:** Primer sequences used in qRT-PCR.

circRNA ID	Primer sequence 5’-3'
hsa_circ_0001492	AACAACGTTACCAGCATCCA
	TCCTCTTCCCCTCGTAGACA
hsa_circ_0000896	CGAGATGCCGACTGATAACTTC
	TCTTGCTTGGAGATGCTGGTA
hsa_circ_0001439	AGTGTGATAATCGAACAACTCCG
	AACAGTCTGCCAGAGTTCCA
GAPDH	GAGTCAACGGATTTGGTCGT
	GAGTCAACGGATTTGGTCGT

F stands for forward; R stands for reverse.

### Detection of Serum CEA and NSE

Serum CEA and NSE concentrations in LUAD patients and healthy controls were determined using the Cobase 602 system with the Elecsys Assay kit (Roche Diagnostics, Basel, Switzerland) according to the manufacturer’s instructions. The cut-off values for CEA and NSE were 5 ng/mL and 16.3 ng/mL, respectively.

### Statistical Analysis

Statistical analysis was performed using the Statistical Program for Social Sciences (SPSS) 22.0 software (SPSS, Chicago, IL, USA) and GraphPad Prism 5.0 (GraphPad Software, La Jolla, CA, USA). The Mann-Whitney unpaired or paired t-test was used to analyze qRT‐PCR results between the case and control groups. Differences between circRNA expression and the clinical indexes were determined using the Chi-square or Fisher’s exact tests, as appropriate. The receiver operating characteristic (ROC) curve and the area under the curve (AUC) were performed to evaluate the diagnostic value of circRNAs. The Youden index was used to calculate the optimal cut-off value of the circRNAs. Statistical significance was considered at P <0.05.

## Results

### Characterization of Serum Exosomes

**Figure 1:**
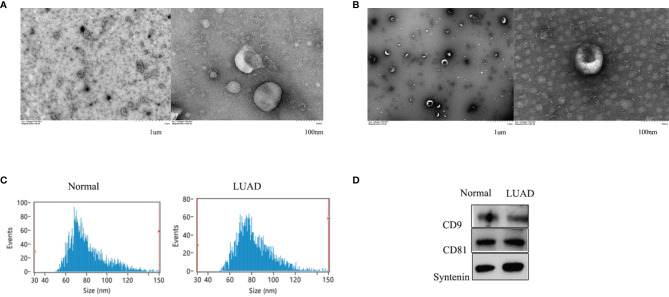
The shapes of serum exosomes from healthy controls **(A)** and LUAD patients **(B)** were photographed by transmission electron microscopy. Size analyses of serum exosomes from healthy controls and LUAD patients were performed by nanoparticle tracking analysis **(C)**. The exosomal markers CD9, CD63, and Syntenin were measured by Western blot **(D)**.

Exosomes isolated from healthy control and LUAD patient serum samples were characterized and confirmed as having a typical size of 30-150 nm and a round membrane-bound morphology (**Figures 1A, B**). The specific exosome diameters were 81.26 and 82.25 nm in the healthy controls and LUAD patients, respectively (**Figure 1C**). Exosomes isolated from the serum were positive for the common exosomal markers, CD9, CD63, and syntenin (**Figure 1D**). These results verified the efficiency and purity of exosome extraction needed for further analysis of the exosomal cicrRNAs.

### Serum and Serum Exosomal CircRNA Expression in LUAD Patients

**Figure 2:**
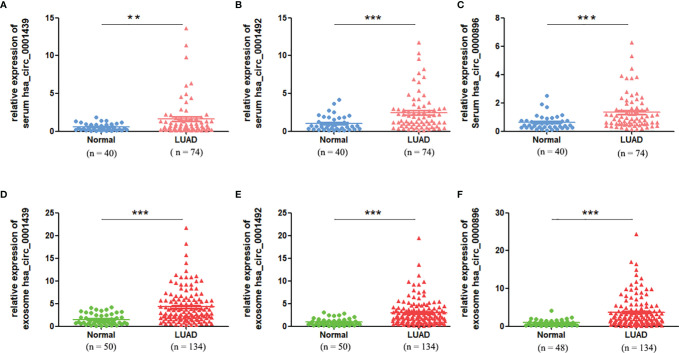
The serum expression levels of has_circ_0001439 **(A)**, hsa_circ_0001492 **(B)**, and hsa_circ_0000896(C) in LUAD (n=74). The serum exosomal expression levels of hsa_circ_0001439 **(D)**, hsa_circ_0001492 **(E)**, and hsa_circ_0000896 **(F)** in LUAD (n=134). (**P < 0.01, ***P < 0.001).

Serum expression of the three circRNAs, hsa_circ_0001439, hsa_circ_0001492, and hsa_circ_0000896, was significantly higher in the LUAD patient group than in the healthy control group (**Figures 2A– C**). QRT-PCR results showed that the levels of serum exosomal hsa_circ_0001439, hsa_circ_0001492, and hsa_circ_0000896 were also significantly higher in the LUAD group than in the healthy control group (**Figures 2D– F**). Taken together, these results indicated that the three circRNAs were enriched in both serum and serum-derived exosomes from LUAD patients.

### Diagnostic Performance of Serum and Serum Exosomal CircRNAs in LUAD Patients

**Figure 3:**
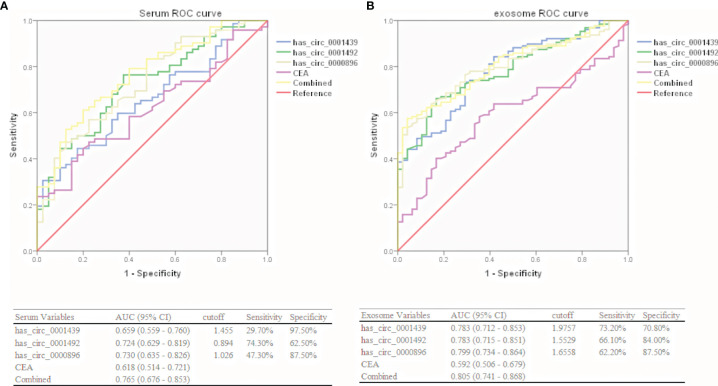
Diagnostic performances of serum **(A)** and serum exosomal **(B)** circRNAs in LUAD patients.

Hsa_circ_0001439, hsa_circ_0001492, and hsa_circ_0000896 were assessed as potential circRNA biomarker candidates for LUAD in comparison with a traditional tumor marker (CEA). The areas under the ROC curve (AUCs) of the three serum circRNAs were 0.659, 0.724, and 0.730, respectively, in the LUAD group, as compared to only 0.618 in the CEA group (**Figure 3A**). In the serum exosome group, the AUC was highest for hsa_circ_0000896 (AUC=0.799), followed by hsa_circ_0001439 (AUC=0.783), hsa_circ_0001492 (AUC=0.783), and CEA (AUC=0.592) (**Figure 3B**). Interestingly, the AUCs of the combined circRNAs, hsa_circ_0001439, hsa_circ_0001492, and hsa_circ_0000896, increased to 0.765 and 0.805 in the serum and serum exosome groups, respectively.

Collectively, these results showed that hsa_circ_0001439, hsa_circ_0001492, and hsa_circ_0000896 had better diagnostic values than CEA. In addition, the AUC values of serum exosomal hsa_circ_0001439, hsa_circ_0001492, and hsa_circ_000089 had greater values for LUAD diagnosis than the serum circRNAs.

### Correlation Between Serum and Serum Exosomal CircRNA Expression and Clinicopathological Factors

LUAD patients were separately divided into two groups with high or low expression of hsa_circ_0001439, hsa_circ_0001492, and hsa_circ_0000896, according to their optimal cut-off values (**Figure 3**). Serum hsa_circ_0001439, hsa_circ_0001492, and hsa_circ_0000896 expression did not correlate with gender, age, pathological grading, diameter, or other traditional tumor markers, such as CEA and NSE (**Table 2**). However, expression of serum exosomal hsa_circ_0001439, hsa_circ_0001492, and hsa_circ_0000896 were significantly associated with pathological grading (**Table 3**). The expression of serum exosomal hsa_circ_0001492 was also associated with tumor diameter and other traditional tumor markers, such as CEA and NSE. Taken together, these data indicated that serum exosomal, but not serum hsa_circ_0001439, hsa_circ_0001492, or hsa_circ_0000896, correlated with the clinicopathologic status of LUAD patients.

**Table 2:** The correlation of serum hsa_circ_0001439, hsa_circ_0001492, and hsa_circ_0000896 expression levels with clinicopathological factors.

Variables	Total	has_circ_0001439		P	Total	has_circ_0001492		P	Total	has_circ_0000896		P
	74	Low(<1.455)	High(≥1.455)		74	Low(<0.894)	High(≥0.894)		74	Low(<1.026)	High(≥1.026)	
Gender				0.7996				0.7778				0.2218
Male		24	9			9	24			20	13	
Female		28	13			10	31			19	22	
Age(yr)				0.9907				0.1104				0.0839
<60		33	10			14	29			19	24	
≥60		19	12			5	26			20	11	
Smoke								0.8298				0.1981
Yes		6	0	0.1706		2	4			5	1	
No		41	19			17	43			29	31	
TNM stage				0.8740				0.8000				0.3753
I		40	18			15	43			29	29	
II+III+IV		12	4			4	12			10	6	
pT stage				0.8264				0.9488				0.3350
T1		42	18			16	44			30	30	
T2+T3+T4		10	4			3	11			9	5	
pN stage				0.8740				0.8000				0.7482
0		40	18			15	43			30	28	
≥1		12	4			4	12			9	7	
pM stage				0.8073				0.9097				0.1886
0		42	19			16	45			30	31	
≥1		10	3			3	10			9	4	
Diameter				0.2424				0.1650				1.0000
<3		22	20			15	47			31	31	
≥3		3	0			2	1			2	1	
NSE				0.2479				0.7836				0.2951
<16.3ng/mL		19	11			6	24			16	14	
≥16.3ng/mL		15	4			4	15			13	6	
CEA				0.8441				0.8596				0.0695
<5ng/mL		41	18			14	45			28	31	
≥5ng/mL		10	3			4	9			10	3	

**Table 3:** The correlation of serum exosomal hsa_circ_0001439, hsa_circ_0001492, and hsa_circ_0000896 expression levels with clinicopathological factors.

Variables	Total	has_circ_0001439		P	Total	has_circ_0001492		P	Total	has_circ_0000896		P
	134	Low(<1.976)	High≥1.976)		134	Low(<1.553)	High≥1.553)		134	Low(<1.656)	High≥1.656)	
Gender				0.6782				0.3385				0.9956
Male		13	35			19	29			19	29	
Female		25	61			27	59			34	52	
Age(yr)				0.9907				0.0737				**0.0292**
<60		23	58			23	58			26	55	
≥60		15	38			23	30			27	26	
Smoke				0.3215				0.2932				0.5006
Yes		5	6			6	5			6	5	
No		29	81			37	73			43	67	
TNM stage				**< 0.0001**				**0.0002**				**0.0351**
I		22	86			29	79			38	70	
II+III+IV		16	10			17	9			15	11	
pT stage				**0.0064**				**0.0011**				0.2249
T1		25	83			30	78			40	68	
T2+T3+T4		13	13			16	10			13	13	
pN stage				**0.0005**				**0.0075**				**0.0404**
0		25	87			33	79			40	72	
≥1		13	9			13	9			13	9	
pM stage				**0.0045**				**0.0052**				**0.0455**
M0		29	91			36	84			44	76	
M1		9	5			10	4			9	5	
Diameter				0.1647				**0.0311**				0.6893
<3		23	74			29	68			35	62	
≥3		6	7			8	5			6	7	
NSE				0.1868				**0.0391**				0.1229
<16.3ng/mL		20	63			27	56			32	51	
≥16.3ng/mL		6	11			9	8			10	7	
CEA				0.1006				**0.0051**				0.0655
<5ng/mL		27	83			33	77			39	71	
≥5ng/mL		8	9			11	6			10	7	

Considering that statistical significance was considered at P <0.05, the bold values with <0.05 were provided in Table 3.

### Serum and Serum Exosomal CircRNA Expression in Preoperative and Postoperative LUAD Patients

**Figure 4:**
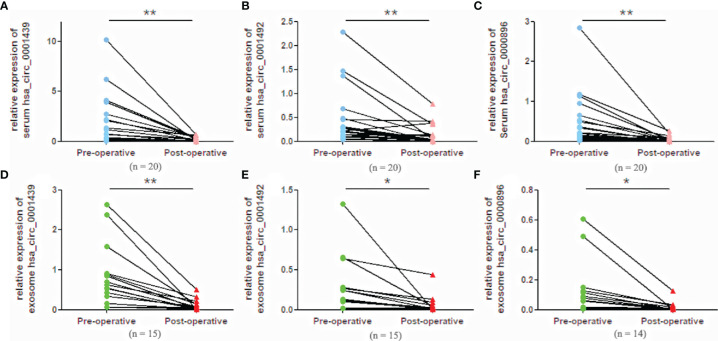
Serum expression levels of hsa_circ_0001439 **(A)**, hsa_circ_0001492 **(B)**, and hsa_circ_0000896 **(C)** compared in preoperative and postoperative LUAD patients. Serum exosomal expression levels of hsa_circ_0001439 **(D)**, hsa_circ_0001492 **(E)**, and hsa_circ_0000896 **(F)** compared in preoperative and postoperative LUAD patients (n = 15). (*P < 0.05, **P < 0.01).

Serum hsa_circ_0001439, hsa_circ_0001492, and hsa_circ_0000896 expressions were statistically higher in preoperative than in postoperative samples (**Figures 4A– C**). Similarly, serum exosomal hsa_circ_0001439, hsa_circ_0001492, and hsa_circ_0000896 expressions were significantly lower following resection of the primary tumor (**Figures 4D–F**).

### Diagnostic Performance of Serum and Serum Exosomal CircRNAs in Preoperative and Postoperative LUAD Patients

**Figure 5:**
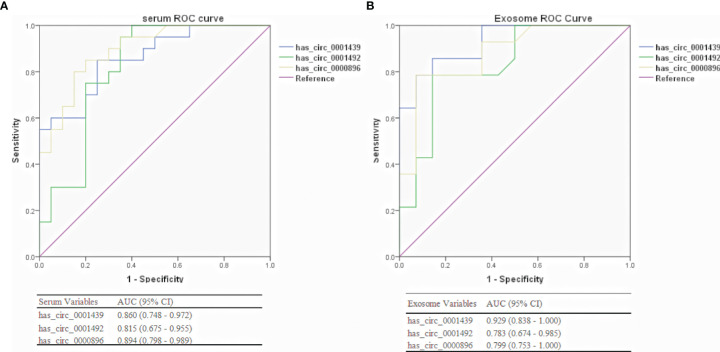
Diagnostic performances of serum **(A)** and serum exosomal **(B)** circRNAs in preoperative and postoperative LUAD patients.

ROC curve analysis was used to confirm the ability of serum and serum exosomal hsa_circ_0001439, hsa_circ_0001492, and hsa_circ_0000896 to serve as biomarkers and distinguish between preoperative and postoperative patients. The AUCs of hsa_circ_0001439 and hsa_circ_0001492 were 0.929 and 0.829, respectively, in serum exosomes and 0.860 and 0.815, respectively, in serum (**Figure 5**). Meanwhile, the AUC of hsa_circ_0000896 was 0.894 and 0.880 in serum and serum exosomes, respectively. These findings indicated that serum and serum exosomal hsa_circ_0001439, hsa_circ_0001492, and hsa_circ_0000896 may be promising markers for effectively monitoring LUAD progression.

## Discussion

LUAD is the most common type of lung cancer and is characterized by high malignancy and poor prognosis. Serum tumor markers such as CEA and NSE are limited by their low sensitivity and specificity (3). Thus, there is a critical need for sensitive, specific, and noninvasive biomarkers to detect and treat LUAD. Liquid biopsy is a non-invasive method that uses bodily fluids including blood, plasma, serum, and exosomes to assess the disease state (14). To date, substantial attention has focused on identifying and quantifying biomarkers, especially circRNAs, in fluids and tissues obtained from tumor biopsies (15, 16). Circulating circRNAs are present in the serum in two forms: serum circRNAs and serum exosomal circRNAs (17). They play an important role in tumorigenesis and several circulating circRNA expression signatures are closely related to tumor diagnosis because of their abundance and stability. Huang et al. found that serum hsa_circ_0070354 may represent a novel diagnostic and predictive biomarker for NSCLC (18). Xian J et al. reported that circ_0047921, circ_0056285, and circ_0007761 expression in serum exosomes could help to distinguish NSCLC patients from healthy controls in the Chinese population (19), and Chen et al. found that serum and serum exosomal hsa_circ_0069313 could discriminate between NSCLC and benign lung tumors (20). These studies show that serum and serum exosomal circRNAs are reliable biomarkers for the occurrence and development of NSCLC.

Our previous study found altered exosomal circRNA expression in LUAD patients and qRT‐PCR results confirmed that some exosomal circRNAs, including hsa_circ_0001439, hsa_circ_0001492, and hsa_circ_0000896, were consistent with sequencing data, which supported their role as potential biomarkers (13). In the current study, the serum and serum-derived exosomal expression and diagnostic value of hsa_circ_0001439, hsa_circ_0001492, and hsa_circ_0000896 were assessed in LUAD patients. Expression of all three circRNAs was significantly higher in LUAD patients than in healthy donors and was significantly decreased after tumor surgical resection, indicating that their levels correlated closely with the presence of tumors. Individual serum and serum exosomal hsa_circ_0001439, hsa_circ_0001492, and hsa_circ_0000896 also exhibited relatively high discriminatory power for identifying LUAD. Findings indicated that serum and serum exosomal hsa_circ_0001439, hsa_circ_0001492, and hsa_circ_0000896 have a promising predictive capacity and could serve as novel diagnostic biomarkers for this disease.

Exosomes are extracellular vesicles secreted by almost all cell types (9). Studies indicate that exosomes derived from tumor cells can enter bodily fluids by carrying tumor-specific circRNA and participating in multiple stages of tumor development. CircRNAs are protected by the lipid bilayer of exosomes and are stable in circulation, providing the potential to serve as tumor biomarkers for various cancers (11). Given the elevated expression of the three exosomal circRNAs, hsa_circ_0001439, hsa_circ_0001492, and hsa_circ_0000896, in LUAD, the association between their expression and patient clinical characteristics was assessed. Serum exosomal hsa_circ_0001492, hsa_circ_0001439, and hsa_circ_0000896 were associated with more aggressive LUAD characteristics, including a higher disease stage. Exosomal hsa_circ_0001492 expression was also positively correlated with CEA and NSE levels. Unexpectedly, a correlation between the three serum circRNA levels and clinical characteristics was not observed. In addition, serum exosomal hsa_circ_0001439, hsa_circ_0001492, and hsa_circ_0000896 exhibited higher diagnostic sensitivity and specificity than serum circRNAs, with AUC values >0.75, which are higher than the CEA AUC value. A combination of the three serum exosomal circRNAs had higher diagnostic sensitivity and specificity than a single marker, with an AUC value of 0.805. These findings imply that serum and serum exosome circRNAs are associated with distinct microenvironmental changes in LUAD patients. Serum exosomal hsa_circ_0001439, hsa_circ_0001492, and hsa_circ_0000896 were also superior to the corresponding serum circRNAs in diagnosing LUAD, suggesting that they may be more reliable biomarkers for this disease.

While studies have assessed the role of exosomal circRNAs in cell-to-cell communication, circRNA sources in cancer are not well characterized, and little is known about the mechanism by which circRNAs are sorted into exosomes (12). The primary hypothesis is that circRNAs are selectively sorted into exosomes and produced by tumors (21). Exosomal circRNAs function as miRNA sponges or interact with binding proteins in host cells to regulate multiple cancer-related biological processes (22, 23). This explains why hsa_circ_0001439, hsa_circ_0001492, and hsa_circ_0000896 expression was identified in the serum and serum exosomes of LUAD patients. It is possible that these circRNAs are stored in exosomes, actively secreted into the serum, and taken up by recipient cells. Future studies are required to characterize the specific biological functions of the three exosomal circRNAs.

In summary, this study showed for the first time that serum and serum exosomal hsa_circ_0001439, hsa_circ_0001492, and hsa_circ_0000896 were highly expressed in LUAD patients and could reliably predict diagnostic disease. CircRNA expression was also significantly lower post- than pre-surgery. Thus, serum and serum exosome hsa_circ_0001439, hsa_circ_0001492, and hsa_circ_0000896 are novel promising markers for the diagnosis and evaluation of LUAD patients. Further studies are needed using a wider range of patients and molecular mechanisms to explore the role and potential use of these circRNAs in LUAD diagnostics and treatment.

## Data Availability Statement

The raw data supporting the conclusions of this article will be made available by the authors, without undue reservation.

## Ethics Statement

This study was approved by the responsible committee for human experimentation of Fujian Provincial Hospital (K-2021-040-04). Informed consent was obtained for all individuals.

## Author Contributions

YK, JY, FC, XX, and LC designed and directed this study. YK, JY, and YG performed the experiments. QC and CH were responsible for collecting patients’ serum samples and clinical information. YK, JY, and LC performed the statistical analysis. YK, JY, and YG wrote the manuscript. FC, XX, and LC reviewed and edited the manuscript. All authors contributed to the article and approved the submitted version.

## Funding

This work was supported by Fujian Natural Science Foundation of China (No. 2021J1375, No.2020J05259); Joint Funds for the Innovation of Science and Technology of Fujian province (No.2019Y9025).

## Conflict of Interest

The authors declare that the research was conducted in the absence of any commercial or financial relationships that could be construed as a potential conflict of interest.

## Publisher’s Note

All claims expressed in this article are solely those of the authors and do not necessarily represent those of their affiliated organizations, or those of the publisher, the editors and the reviewers. Any product that may be evaluated in this article, or claim that may be made by its manufacturer, is not guaranteed or endorsed by the publisher.
